# Monetary reward mechanism for promoting online knowledge sharing: A modeling study

**DOI:** 10.1371/journal.pone.0320236

**Published:** 2025-04-07

**Authors:** Xuhui Hu, Yang Qin, Lu-Xing Yang, Xiaofan Yang

**Affiliations:** 1 School of Big Data and Software Engineering, Chongqing University, Chongqing, China; 2 School of Information Technology, Deakin University, Melbourne, Victoria, Australia; Teesside University, UNITED KINGDOM OF GREAT BRITAINAND NORTHERN IRELAND

## Abstract

Knowledge sharing is critical for an organization to acquire sustained competitive advantage. Bestowing monetary rewards may possibly the most direct method of stimulating online knowledge sharing. Under the monetary reward mechanism for promoting knowledge sharing, we intend to find a satisfactory knowledge-sharing promotion policy. First, based on a state evolutionary model for the knowledge-sharing community, we reduce the original problem to an optimal control model. Second, applying optimal control theory to the model, we give an algorithm for solving the model. Next, we validate the feasibility of the algorithm. Finally, we inspect the applicability of the algorithm. To our knowledge, this is the first time the optimal control modeling technique is applied to the research of knowledge sharing.

## Introduction

Knowledge is recognized as critical organizational resource. Knowledge management refers to the entire process from knowledge creation and knowledge sharing to knowledge utilization [1]. Through effective knowledge management, an organization can enhance innovative capability and acquire sustainable competitive advantage [1]. Knowledge sharing, a vital component of the knowledge management process, refers to the exchange of knowledge among organizational employees with the aim of collectively solving work-related problems and improving overall organizational performance. [2]. The integration of digital technologies in organizational processes has transformed knowledge sharing practices from oﬄine to online [3–5]. Online knowledge sharing refers to the practice of exchanging knowledge, expertise, and information through digital platforms, making it possible for employees to engage in collaborative efforts, irrespective of geographic location. Online platforms now serve as essential tools in this transformation, enabling not only remote and instant access but also fostering a more inclusive and persistent knowledge-sharing ecosystem. [6,7]. Online knowledge-sharing communities rely heavily on the voluntary and effortful contributions of employees. The content generated within these communities is often grounded in employees’ expert knowledge and firsthand experience, making it a valuable asset for both individual and organizational growth [8,9]. However, the sustainability of content generation in these communities is largely contingent upon the motivation of contributors. To encourage ongoing participation, organizations increasingly turn to incentive mechanisms, particularly monetary rewards [10,11]. Previous research differentiate rewards into two categories: peer-based, where individuals incentivize peers at personal cost [12], and institutional, where organizations systematically reward contributors [13]. Current research largely presumes that reward policies are static, with the intensity of incentives remaining unchanged despite evolving community dynamics. Although monetary rewards serve as a powerful extrinsic motivator, their effectiveness is highly sensitive to strategic design and adaptive implementation. Poorly structured reward systems may lead to unintended consequences, such as a focus on short-term contributions rather than long-term engagement [14], or a decrease in intrinsic motivation to share knowledge [15]. To address these limitations, our study proposes an open-loop optimal control framework that designs predefined reward policies balancing organizational costs with long-term engagement.

### Motivation

Consider the situation where an organizational employee (she, for short) holds some kind of valuable knowledge (new skills, new methods, etc.) from the sharing of which other organizational employees as well as the organization itself could benefit (the employees’ job performance could be enhanced significantly, the organizational expected performance could be attained, etc.). From organizational standpoint, the sharing of the knowledge is encouraged. But from individual standpoint, the employee faces the choice of sharing her knowledge or withholding her knowledge. This situation is referred to as the *knowledge-sharing dilemma* [16–18]. In this situation, the employee has to carefully trade the benefits of sharing her knowledge (gaining expert status, receiving public praise, etc.) against the cost of doing so (time cost for organizing documents about her knowledge, risk of exposing her competitive edge, risk of being terminated). She only prefers to sharing her knowledge if the estimated total benefit exceeds the estimated total cost. Otherwise, she would choose to withhold her knowledge.

Participants in online knowledge sharing are categorized as lurkers (i.e., those who read posts without making any contribution) and posters (i.e., those who share knowledge through posting) [19,20]. Reportedly, the majority of participants (exceeding 90%) are lurkers [21]. In order to survive and succeed in fierce market competition, organizations should take a variety of measures, ranging from training self-efficacy to bestowing rewards, to motivate their employees’ knowledge sharing intentions [8,9,22].

Among all possible knowledge-sharing promotion mechanisms, awarding monetary rewards may possibly be the most direct promotion mechanism. As far as we are concerned, there has been no report in literature on systematic research of monetary reward mechanism for promoting knowledge sharing. Under the time continuity assumption of the rewards, the present paper addresses the issue. We refer to the function of reward increase rates for all organizational employees at all time points as a knowledge-sharing promotion (KSP) policy. In practice, there exist numerous feasible KSP policies, and different KSP policies lead to different cost benefits. Consequently, it is of practical importance to consider the following problem:

*knowledge-sharing promotion (KSP) problem:* Under the monetary reward mechanism for promoting online knowledge sharing, find a cost-effective KSP policy among all feasible KSP policies.

As far as we are concerned, there is no report in literature on the problem. This paper is devoted to addressing the problem through mathematical modeling.

### Contributions

In this paper the following contributions are made.

- First, we explicitly formulate the KSP problem for the first time. Second, we introduce a mathematical model for characterizing the evolution of state of the knowledge-sharing community. Finally, we model the KSP problem as an optimal control problem (the KSP model).
- First, applying optimal control theory to the KSP model, we give a numerical algorithm for solving the KSP model. Second, we corroborate the feasibility of the algorithm through extensive experiments.
- First, we inspect the applicability of the algorithm by giving estimation/approximation schemes for the relevant parameters/functions. Second, we examine the influence of these factors on the performance of the algorithm.

This paper pioneers the application of optimal control theory in the research of knowledge sharing. The subsequent materials are organized in this fashion: The related work section reviews the related work. The mathematical modeling of the KSP problem section and the solution of the KSP model section establish and solve the KSP model, respectively. The further discussions section makes further discussions. This work is summarized by the concluding remarks section.

## Related work

This section reviews the related work and highlights the innovations of the present paper.

### Knowledge sharing promotion mechanism

The social dilemma of knowledge sharing renders most organizational employees to withhold their valuable knowledge (see Subsection I-A). This condition seriously weakens organizational competitive advantage. For the purpose of promoting knowledge sharing, much efforts have been put on investigating the influence of various factors, ranging from intrinsic factors such as self-efficacy and enjoyment to extrinsic factors such as rewards and reciprocity, on knowledge sharing behavior [23–26]. Previous studies have predominantly focused on posters because of their direct contributions to knowledge sharing [27]. In particular, it was empirically found that external incentives, such as rewards and recognition, are pivotal in motivating posters to contribute actively [28,29]. In recent years, the function of lurkers in knowledge sharing has received considerable interest [8,9,22].

The promotion mechanism for knowledge sharing has been explored extensively. For instance, [16] proposed three feasible approaches to promoting knowledge sharing: reconstructing the pay-off function, increasing efficacy, and promoting group identity and personal responsibility. Selectively rewarding individual contributions in the form of monetary reward or social recognition or collective gains is viewed as an effective means of reconstructing the pay-off function. Establishing a feedback mechanism of using shared knowledge is regarded as a feasible technique of increasing efficacy. Establishing knowledge-sharing community is recognized as an applicable way of promoting group identity. As another example, [18] suggested to improve organizational knowledge-sharing culture by changing the performance climate. [30,31] investigated the cost-effectiveness of institutional incentives for promoting cooperation and proposed mathematical optimization models for reward and punishment mechanisms in the evolution of group cooperation. Building on this foundation, this paper further extends the application scenarios of incentive mechanisms by focusing on the design of dynamic incentive strategies in the process of organizational online knowledge sharing.

To our knowledge, all previous explorations on knowledge sharing promotion mechanism were conducted on a qualitative basis as opposed to a quantitative basis.

### Applications of optimal control theory

Optimal control theory is devoted to finding an optimal control strategy for a dynamic system [32]. In the past decades, optimal control theory has witnessed widespread applications in diverse areas, ranging from containment of malware propagation [33,34] and suppression of rumor spreading [35,36] to defense against advanced cyberattacks [37,38] and development of advertising policy [39,40]. [41,42] explored the incentive strategies in evolutionary cooperation optimization, especially using optimal control theory to optimize incentive strategies to minimize costs when using positive and negative incentives to achieve the target state. Our research extends this framework to the context of online community knowledge sharing, and is dedicated to finding personalized and optimal incentive strategies for different individuals. In particular, [43] is closely related to the present paper. In this paper, the product co-creation problem was resolved through optimal control approach. Here, valuable suggestions about a new product are shared in the company-sponsored online community, somewhat like the situation where valuable knowledge are shared in the online knowledge-sharing community.

As far as we are concerned, there is no report in literature on the application control theory to the research of knowledge sharing.

### Innovations of this work

The innovations of this work are threefold. First, the problem of promoting knowledge sharing through monetary reward (the KSP problem) is proposed. The problem is of practical importance, because its solution helps promote knowledge sharing. Second, by characterizing the evolution of state of the knowledge-sharing community over time, the KSP problem is modeled successfully as an optimal control problem (the KSP model). Finally, the KSP model is resolved successfully.

## Mathematical modeling of the KSP problem

This section is devoted to the mathematical modeling of the KSP problem. First, a KSP policy is formulated mathematically. Second, the evolutionary process of state of the knowledge-sharing community is characterized by a dynamic model. Finally, the KSP problem is modeled mathematically.

### KSP policy

Consider the KSP problem. Suppose the organization intends to create and maintain an online community for facilitating the sharing of knowledge among its employees. We refer to the community as the *knowledge-sharing (KS) community* held by the organization. Suppose the community accommodates all organizational employees (numbered 1 through *N*) as members, each member is encouraged to create posts in the community to share new knowledge, and each member has access to all posts retained in the community.

Suppose the organization intends to promote knowledge sharing through monetary rewards in the time horizon  [ 0 , *T* ] . Here, *t* = 0 stands for the time at which the KS community is created. *T* is termed the *knowledge-sharing promotion period*. For all *i* and *t*, let $x_{i}(t)$ denote the increase rate of the amount of money given to the *i*-th employee at time *t* for exciting her to share her knowledge, which is termed the *i*-th *reward increase rate* at time *t*. Furthermore, the function


$$\begin{array}{ccc} x(t)=(x_{1}(t),\ldots ,x_{N}(t)),\;0\leq t\leq T, & & \end{array}$$ (1)

is termed a *knowledge-sharing promotion (KSP) policy*. In what follows, the KSP policy is abbreviated as *x*.

Skilled employees have rich knowledge. If a skilled employee share her knowledge in the KS community, those employees who acquire the knowledge could significantly enhance their respective job performance. For the purpose of making up a cost-effective KSP policy, the value of the knowledge shared by each employee needs to be estimated based on his historical job performance. On this basis and in view of the limited reward budget, the maximum possible reward increase rate for each employee at any time is determined. Let ${\bar{x}}_{i}$ denote the maximum possible reward increase rate for the *i*-th employee at any time. Let $\bar{x}=({\bar{x}}_{1},\cdots \;,{\bar{x}}_{N})$, which is termed the *maximum reward increase rate* over the KS community. On the other hand, since piecewise continuous functions cover all practically useful functions, in what follows it is reasonably assumed that all KSP policies are piecewise continuous. In summary, we assume the set of feasible KSP policies is


$$\begin{array}{ccc} X=\left\{x\in PC{\left[0,T\right]}^{N}:0\leq x(t)\leq \bar{x},\;0\leq t\leq T\right\}, & & \end{array}$$ (2)

where *PC* [ 0 , *T* ]  stands for the set of piecewise continuous functions on the interval  [ 0 , *T* ] .

Under the KSP policy *x*, the total amount of money paid to all organizational employees equals


$$\begin{array}{ccc} C(x)=\int_{0}^{T}\overset{N}{\underset{i=1}{\sum }}x_{i}(t)dt. & & \end{array}$$ (3)

Eq (3) represents the total cost of monetary rewards provided by the organization to all employees over the time horizon  [0,T]. Here, the summation $\sum_{i=1}^{N}x_{i}(t)$ accounts for the reward increase rates of all employees at the time *t*, and the total cost is the cumulative sum of these rates over the the entire time horizon  [ 0 , *T* ] . This formulation is based on the assumptions that rewards are distributed continuously over time and that the reward increase rates are piecewise continuous functions.

### State of the KS community and its evolution

We refer to an employee as *posting* at a given time if she is creating a post used for sharing her knowledge at the time. Otherwise, we refer to the employee as *lurking* at the time. For all *i* and *t*, let $P_{i}(t)$ (resp. $L_{i}(t)$) denote the probability of the *i*-th employee being posting (resp. lurking) at time *t*. Then, $L_{i}(t)=1-P_{i}(t)$. The vector


$$\begin{array}{ccc} P(t)=(P_{1}(t),\cdots \;,P_{N}(t)) & & \end{array}$$ (4)

is termed the *state of the KS community* at time *t*. Since the initial states of all employees can be observed based on their prior participation performance, we have $P(0)=P_{0}$.

The state of the KS community varies over time. To capture the evolution of state of the KS community over time, introduce a collection of notations and assumptions.

First, owing to the exciting effect of monetary reward, each lurker may possibly become posting at any time. In what follows it is reasonably assumed that, owing to the the exciting effect of monetary reward, the *i*-th employee becomes posting from lurking at time *t* at rate $f(x_{i}(t))$. Here, *f* ( 0 ) = 0, *f* is increasing and flattens out (the exciting effect of monetary reward slows down). In what follows, *f* is termed the *reward influence function*.

Second, owing to the exciting effect of posters, each lurker may potentially become a poster at any time. In what follows it is reasonably assumed that, owing to the exciting effect of posters, the *i*-th employee becomes posting from lurking at time *t* at rate $g\left(\sum_{j\neq i}P_{j}(t)\right)$. Here, *g* ( 0 ) = 0, the function *g* is increasing and flattens out (the exciting effect of posters slows down). The term $g\left(\sum_{j\neq i}P_{j}(t)\right)$ captures peer influence: the summed posting probabilities $\left(\sum_{j\neq i}P_{j}(t)\right)$ represent community-wide activity, which incentivizes lurkers to participate. The function *g* increasing yet saturating, reflects diminishing marginal returns of group engagement. In what follows, *g* is termed the *poster influence function*. For ease in mathematical treatment, in what follows assume *g* is differential.

Finally, owing to a variety of reasons (busyness with work, rest, etc.), each employee becomes lurking from posting at any time at the constant positive rate *α*, which is termed the *lurking rate*.

In summary, under the KSP policy *x* the state of the KS community evolves following the differential system


$$\begin{array}{ccc} \{\begin{array}{ccc} & \frac{dP_{i}(t)}{dt}=-\alpha P_{i}(t)+\left[f\left(x_{i}(t)\right)+g\left(\underset{j\neq i}{\sum }P_{j}(t)\right)\right] & \\ & \;\times \left[1-P_{i}(t)\right],\;0\leq t\leq T,i=1,\cdots \;,N,\\ & P(0)=P_{0}. \end{array} & & \end{array}$$ (5)

**Fig 1:**
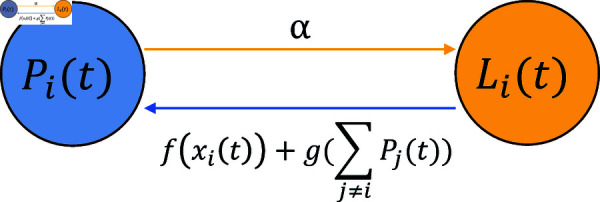
Illustration of the evolutionary model, describing the state evolution of the KS community.

The system is termed the *evolutionary model* for the state of the KS community. See Fig 1 for an illustration of the model.

### Optimal control modeling of the KSP problem

Every poster can bring benefit to the organization. For *i* = 1 , ⋯ , *N*, let $\omega_{i}$ denote the benefit per unit time generated by the *i*-th employee if she is posting. Let $\omega =(\omega_{1},\cdots \;,\omega_{N})$. In what follows it is reasonably assumed that *ω* is linearly proportional to $\bar{x}$, i.e., $\omega =c\bar{x}$, where *c* is a positive constant, which is termed the *reward-return coefficient*. Under the KSP policy *x*, the overall benefit gained by the organization is estimated to be


$$\begin{array}{ccc} B(x)=c\int_{0}^{T}\overset{N}{\underset{i=1}{\sum }}{\bar{x}}_{i}P_{i}(t)dt. & & \end{array}$$ (6)

In view of the cost needed for executing the KSP policy *x* (see Eq (2)), the cost benefit of executing the KSP policy *x* is estimated to be


$$\begin{array}{ccc} J(x)=c\int_{0}^{T}\overset{N}{\underset{i=1}{\sum }}{\bar{x}}_{i}P_{i}(t)dt-\int_{0}^{T}\overset{N}{\underset{i=1}{\sum }}x_{i}(t)dt. & & \end{array}$$ (7)

Therefore, the KSP problem can be modeled as the optimal control problem


max ⁡ x∈XJ(x)=c∫ 0T ∑i=1Nx¯iPi(t)dt−∫ 0T ∑i=1Nxi(t)dts.t. {dPi(t)dt=−αPi(t)+ [f (xi(t))+g (∑j≠iPj(t))]× [1−Pi(t)],0≤t≤T,i=1,⋯N,P(0)=P0. (8)

We refer to the optimal control problem as the *KSP model*.

Each instance of the KSP model (8) is represented by the 8-tuple


$$\begin{array}{ccc} M=(N,T,\bar{x},f,g,\alpha ,c,P_{0}). & & \end{array}$$ (9)

For convenience, in what follows each instance of the KSP model is termed a *KSP instance*.

## A solution of the KSP model

This section is committed to solving the KSP model established in the preceding section.

### An algorithm for solving the KSP model

The Hamiltonian function for the KSP model (8) reads


$$\begin{array}{ccc} \begin{array}{ccc} & \;H(P,x,\lambda )=c\overset{N}{\underset{i=1}{\sum }}{\bar{x}}_{i}P_{i}-\overset{N}{\underset{i=1}{\sum }}x_{i}-\alpha \overset{N}{\underset{i=1}{\sum }}\lambda_{i}P_{i} & \\ & \;+\overset{N}{\underset{i=1}{\sum }}\lambda_{i}(1-P_{i})\left[f(x_{i})+g\left(\underset{j\neq i}{\sum }P_{j}\right)\right] \end{array} & & \end{array}$$ (10)

Here, $\lambda =(\lambda_{1},\lambda_{2},...,\lambda_{N})$ stands for the adjoint.

Suppose *x* is an optimal control for the KSP model (8). Let *P* be the solution to the corresponding state evolutionary model (5). Pontryagin Maximum Principle [32] tells us that there exists an adjoint function *λ* such that *λ* ( *T* ) = 0 and


$$\begin{array}{ccc} \frac{d\lambda_{i}(t)}{dt}=-\frac{\partial H(P(t),x(t),\lambda (t))}{\partial P_{i}},\;0\leq t\leq T,i=1,\cdots \;,N. & & \end{array}$$ (11)

Direct calculations lead to


$$\begin{array}{ccc} \{\begin{array}{ccc} & \frac{d\lambda_{i}(t)}{dt}=-c{\bar{x}}_{i}+\lambda_{i}(t)\left[\alpha +f(x_{i}(t))+g\left(\underset{j\neq i}{\sum }P_{j}(t)\right)\right] & \\ & \;-\overset{N}{\underset{j\neq i}{\sum }}\lambda_{j}(t)[1-P_{j}(t)]g^{\prime }\left(\underset{k\neq j}{\sum }P_{k}(t)\right)\\ & \;0\leq t\leq T,i=1,\cdots \;,N,\\ & \lambda (T)=0. \end{array} & & \end{array}$$ (12)

Again by Pontryagin Maximum Principle [32], we get that


x(t)∈ arg ⁡ max ⁡ 0≤y≤x¯H(P(t),y,λ(t)),0≤t≤T. (13)

Simple algebra leads to


xi(t)∈ arg ⁡ max ⁡ 0≤z≤x¯i {λi(t)[1−Pi(t)]f(z)−z},0≤t≤T,i=1,⋯,N. (14)

By combining the system (5), the system (12), and the system (14), we get the optimality system for the KSP problem, which is shown in the system (15). In what follows, the optimality system is viewed as a system in the KSP polocy *x*, whereas the state function *P* and the adjoint function *λ* are viewed as auxiliary functions.


 {dPi(t)dt=−αPi(t)+ [f (xi(t))+g (∑j≠iPj(t))] [1−Pi(t)],0≤t≤T,i=1,⋯,N,dλi(t)dt=−cx¯i+λi(t) [α+f(xi(t))+g (∑j≠iPj(t))]−∑j≠iNλj(t)[1−Pj(t)]g′ (∑k≠jPk(t)),0≤t≤T,i=1,⋯,N,xi(t)∈arg ⁡ max ⁡ 0≤z≤x¯i {λi(t)[1−Pi(t)]f(z)−z},0≤t≤T,i=1,⋯,N,P(0)=P0,λ(T)=0, (15)

By applying the Forward-Backward Sweep Method [44] to the optimality system (15), we get a numerical algorithm for solving the KSP model (8), which we refer to as the *KSP algorithm*. See Algorithm 1 for a pseudo-code description of the algorithm. The basic idea for the KSP Algorithm is to generate a sequence of feasible KSP policies by iteratively solving the optimality system (15) until the generated sequence converges. Then, the KSP algorithm outputs the finally generated KSP policy.

**Algorithm 1**: KSP

**Input:** KSP instance $M=(N,T,\bar{x},f,g,\alpha ,c)$,convergence error *ϵ*.

**Output:** KSP policy *x*.

1:   *k* ← 0; $x^{\left(0\right)}\leftarrow \bar{x}$;

2:   **repeat**

3:    *k* ← *k* + 1;

4:    calculate the state function *P* using the system (5) with $x\leftarrow x^{\left(k-1\right)}$;

5:    $P^{k}\leftarrow P$;

6:    calculate the adjoint function *λ* using the system (12) with $x\leftarrow x^{\left(k-1\right)}$ and $P^{k}\leftarrow P$;

7:    $\lambda^{k}\leftarrow \lambda$;

8:    calculate the KSP policy *x* using the system (14) with $P\leftarrow P^{k}$, and $\lambda \leftarrow \lambda^{k}$;

9:    $x^{\left(k\right)}\leftarrow x$;

10:   **until** max ⁡ 1≤i≤N sup ⁡ 0≤t≤T |xi(k)(t)−xi(k−1)(t)|<ϵ; **return** $x^{\left(k\right)}$.

### Feasibility of the KSP algorithm

We refer to the KSP algorithm as feasible if (i) the algorithm converges, i.e., the sequence of KSP policies generated by the KSP algorithm converges, and (ii) the algorithm is satisfactory, i.e., the KSP policy returned by the KSP algorithm beats the majority of other feasible KSP policies (in terms of the cost benefit). Now, validate the feasibility of the KSP algorithm through numerical experiments.

**Experiment 1.** *Consider the KSP instance*


$$M_{1}=(N,T,\bar{x},f,g,\alpha ,c,P_{0}).$$

*Here,* *N* = 100, *T* = 3, $\bar{x}$ *is a vector generated randomly in the region* $\prod_{i=1}^{N}(0,{\bar{x}}_{i})$, *f* ( *z* ) = 0 . 3*tanh* ⁡  ( *z* ) , *g* ( *z* ) = 0 . 2*ln* ⁡  ( 1 + *z* ) , *α* = 0 . 1, *c* = 10, *and* $P_{0}$ *is a vector generated randomly within the interval*  [ 0 , 0 . 5 ] .

**Fig 2:**
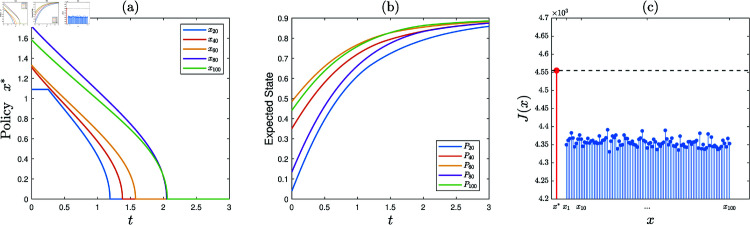
Experiment 1 is conducted with the KSP instance $M_{1}=(N,T,\bar{x},f,g,\alpha ,c,P_{0})$, where *N* = 100, *T* = 3, $\bar{x}$ is a vector generated randomly in the region $\prod_{i=1}^{N}(0,{\bar{x}}_{i})$, *f* ( *z* ) = 0 . 3*tanh* ⁡  ( *z* ) , *g* ( *z* ) = 0 . 2*ln* ⁡  ( 1 + *z* ) , *α* = 0 . 1, *c* = 10, and $P_{0}$ is a vector generated randomly within the interval  [ 0 , 0 . 5 ] . After executing the KSP algorithm, we obtain: (a) the KSP policy $x^{*}$, (b) expected employee state evolution over time, (c) *J*(*x*), $x\in \{x^{*}\}\cup S$.

1) *Running the KSP algorithm on* $\left(M_{1},10^{-6}\right)$, *it is observed that the generated sequence of KSP policies converges. Let* $x^{*}$ *denote the finally generated KSP policy. See Fig 2(a) for partial display of* $x^{*}$ *(the full data is available in Supporting information)*.
2) *Generate a set of 100 feasible KSP policies randomly and uniformly, denoted* $S=\{x^{1},\cdots \;,x^{100}\}$. *Fig 2(b) exhibits* *J*(*x*), $x\in \{x^{*}\}\cup S$ *(the full data is available in Supporting information). It is observed that* $J(x^{*})>J(x)$, *x* ∈ *S*. *Hence,* $x^{*}$ *is satisfactory*.

**Experiment 2.** *Consider the KSP instance*


$$M_{2}=(N,T,\bar{x},f,g,\alpha ,c,P_{0}).$$

*Here*, *N* = 300, *T* = 4, $\bar{x}$ is a vector generated randomly in the region $\prod_{i=1}^{N}(0,{\bar{x}}_{i})$, *f* ( *z* ) = 0 . 4*tanh* ⁡  ( *z* ) , *g* ( *z* ) = 0 . 1*ln* ⁡  ( 1 + *z* ) , *α* = 0 . 05, *c* = 5, and $P_{0}$ is a vector generated randomly within the interval  [ 0 , 0 . 5 ] .

**Fig 3:**
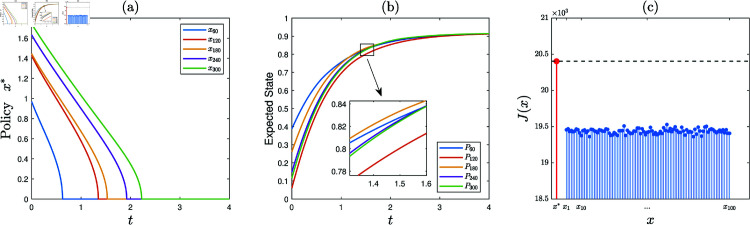
Experiment 2 is carried out with the KSP instance $M_{2}=(N,T,\bar{x},f,g,\alpha ,c,P_{0})$, where *N* = 300, *T* = 4, $\bar{x}$ is a vector generated randomly in the region $\prod_{i=1}^{N}(0,{\bar{x}}_{i})$, *f* ( *z* ) = 0 . 4*tanh* ⁡  ( *z* ) , *g* ( *z* ) = 0 . 1*ln* ⁡  ( 1 + *z* ) , *α* = 0 . 05, *c* = 5, and $P_{0}$ is a vector generated randomly within the interval  [ 0 , 0 . 5 ] . After leveraging the KSP algorithm, we get: (a) the KSP policy $x^{*}$, (b) expected employee state evolution over time, (c) *J*(*x*), $x\in \{x^{*}\}\cup S$.

1) Running the KSP algorithm on $\left(M_{2},10^{-6}\right)$, it is observed that the generated sequence of KSP policies converges. Let $x^{*}$ denote the finally generated KSP policy. See Fig 3(a) for partial exhibition of $x^{*}$ (the full data is available in Supporting information).
2) Generate a set of 100 feasible KSP policies randomly and uniformly, denoted $S=\{x^{1},\cdots \;,x^{100}\}$. Fig 3(b) demonstrates *J*(*x*), $x\in \{x^{*}\}\cup S$ (the full data is available in Supporting information). It is observed that $J(x^{*})>J(x)$, *x* ∈ *S*. Hence, $x^{*}$ is satisfactory.

**Experiment 3.** *Consider the KSP instance*


$$M_{3}=(N,T,\bar{x},f,g,\alpha ,c,P_{0}).$$

*Here*, *N* = 500, *T* = 5, $\bar{x}$ is a vector generated randomly in the region $\prod_{i=1}^{N}(0,{\bar{x}}_{i})$, *f* ( *z* ) = 0 . 3*tanh* ⁡  ( *z* ) , *g* ( *z* ) = 0 . 2*ln* ⁡  ( 1 + *z* ) , *α* = 0 . 1, *c* = 10, and $P_{0}$ is a vector generated randomly within the interval  [ 0 , 0 . 5 ] .

**Fig 4:**
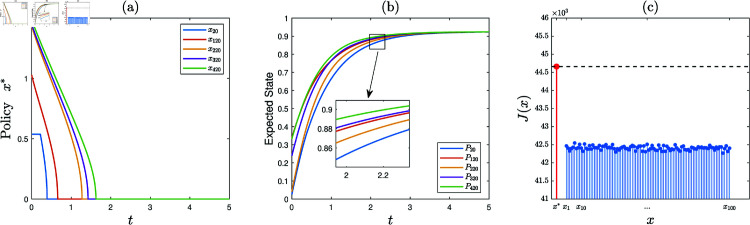
Experiment 3 is performed with the KSP instance $M_{3}=(N,T,\bar{x},f,g,\alpha ,c,P_{0})$, where *N* = 500, *T* = 5, $\bar{x}$ is a vector generated randomly in the region $\prod_{i=1}^{N}(0,{\bar{x}}_{i})$, *f* ( *z* ) = 0 . 3*tanh* ⁡  ( *z* ) , *g* ( *z* ) = 0 . 2*ln* ⁡  ( 1 + *z* ) , *α* = 0 . 1, *c* = 10, and $P_{0}$ is a vector generated randomly within the interval  [ 0 , 0 . 5 ] . After executing the KSP algorithm, we obtain: (a) the KSP policy $x^{*}$, (b) expected employee state evolution over time, (c) *J*(*x*), $x\in \{x^{*}\}\cup S$.

1) Running the KSP algorithm on $\left(M_{3},10^{-6}\right)$, it is observed that the generated sequence of KSP policies converges. Let $x^{*}$ denote the finally generated KSP policy. See Fig 4(a) for partial demonstration of $x^{*}$ (the full data is available in Supporting information).
2) Generate a set of 100 feasible KSP policies randomly and uniformly, denoted $S=\{x^{1},\cdots \;,x^{100}\}$. Fig 4(b) demonstrates *J*(*x*), $x\in \{x^{*}\}\cup S$ (the full data is available in Supporting information). It is observed that $J(x^{*})>J(x)$, *x* ∈ *S*. Hence, $x^{*}$ is satisfactory.

Based on 1000 similar experiments, we conclude that (i) the generated sequence of KSP policies converges and (ii) the finally generated KSP policy is satisfactory in terms of the cost benefit. As thus, we conclude that the KSP algorithm is feasible. Therefore, we recommend the KSP algorithm.

The computational efficiency of the KSP algorithm primarily depends on the number of employees *N*, Our experiments demonstrate that the algorithm converges efficiently for moderate-sized problems(e.g., *N* = 100 , 300 , 500 and *T* = 3 , 4 , 5). However, for very large *N*, the computational cost may increase significantly due to the larger system size. The algorithm requires solving a set of differential equations for each employee. As *N* increases, the size of the system also increases, leading to higher computational complexity. This is particularly evident in the state evolutionary model (Eq 5) and the adjoint system (Eq 12), both of which involve *N* equation. Additionally, in rare scenarios (e.g., initial guesses far from the optimal solution, highly nonlinear reward or poster influence functions), the algorithm may require more iterations or parameter adjustments to ensure convergence.

## Further discussions

In the preceding section, the KSP algorithm was proposed and its feasibility was corroborated. This section addresses some issues related to the KSP algorithm. First, the applicability of the KSP algorithm is inspected. Second, the influence of some factors is examined.

### Applicability of the KSP algorithm

First, consider the applicability of the KSP algorithm. There are seven factors involved in the KSP instance (9): the number of organizational employees, *N*, the knowledge-sharing promotion period, *T*, the maximum reward increase rate, $\bar{x}$, the reward influence function, *f*, the poster influence function, *g*, the lurking rate, *α*, and the reward-return coefficient. Before the KSP algorithm can be applied to a real-world scenario, all the seven relevant factors need to be determined or estimated or approximated.

The number of organizational employees is already known. The knowledge-sharing promotion period is determined by the demand on knowledge sharing. The maximum reward increase rate is determined by the scheduled reward budget as well as each employee’s historical job performance. The lurking rate and the reward-return coefficient are estimated by the organization through collecting and processing relevant historical data. The reward influence function and the poster influence function are approximated by the organization through collecting and fitting relevant historical data. Once all the factors involved in the KSP instance are determined, the KSP algorithm can be applied to the KSP instance to generate a cost-effective KSP policy.

### Influence of some factors

First, inspect the influence of some factors on the performance of the KSP algorithm. First, examine the influence of the number of employees.

**Experiment 4.** *Let *N* = { 10 , 20 , ⋯ , 100 } . Consider the KSP instances*


$$M_{N}=(N,T,\bar{x},f,g,\alpha ,c,P_{0}),\;N\in N.$$

**Fig 5:**
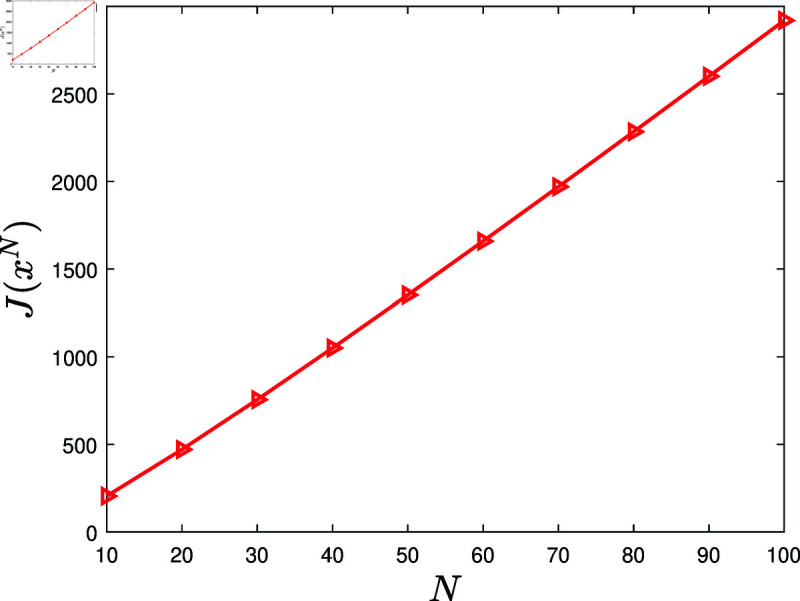
Experiment 4 is conducted with the KSP instance $M_{N}=(N,T,\bar{x},f,g,\alpha ,c,P_{0})$, where *N* ∈ *N*, *T* = 5, $\bar{x}$ is a vector generated randomly in the region $\prod_{i=1}^{N}(0,{\bar{x}}_{i})$, *f* ( *z* ) = 0 . 3*tanh* ⁡  ( *z* ) , *g* ( *z* ) = 0 . 2*ln* ⁡  ( 1 + *z* ) , *α* = 0 . 05, *c* = 5, and $P_{0}$ is a zero vector. This figure exhibits $J(x^{N})$ versus *N*, *N* ∈ *N.*

*For *N* ∈ *N*, let $x^{N}$ denote the KSP policy generated by running the KSP algorithm on $\left(M_{N},10^{-6}\right)$. Fig 5 displays $J(x^{N})$, *N* ∈ *N*. It is observed that $J(x^{N})$ is increasing with the increasing *N*.*

Based on 100 similar experiments, we conclude that the performance of the KSP algorithm is increasing with the increasing number of employees. This conclusion implies that the recommended KSP policy is especially suited to large-sized organizations.

second, examine the influence of the knowledge-sharing promotion period.

**Experiment 5.** *Let *T* = { 1 , 2 , ⋯ , 10 } . Consider the KSP instances*


$$M_{T}=(N,T,\bar{x},f,g,\alpha ,c,P_{0}),\;T\in T.$$

**Fig 6:**
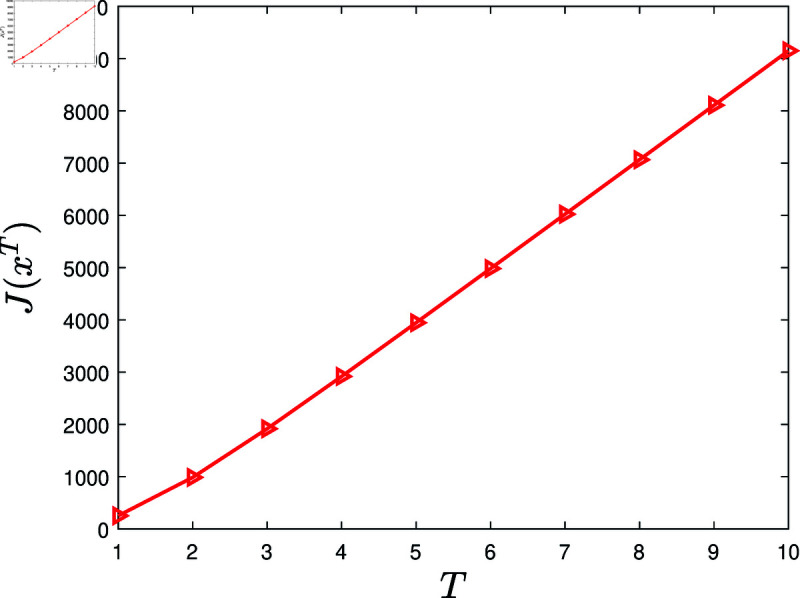
Experiment 5 is carried out with the KSP instance $M_{T}=(N,T,\bar{x},f,g,\alpha ,c,P_{0})$, where *N* = 100, *T* ∈ *T*, $\bar{x}$ is a vector generated randomly in the region $\prod_{i=1}^{N}(0,{\bar{x}}_{i})$, *f* ( *z* ) = 0 . 3*tanh* ⁡  ( *z* ) , *g* ( *z* ) = 0 . 2*ln* ⁡  ( 1 + *z* ) , *α* = 0 . 05, *c* = 5, and $P_{0}$ is a vector generated randomly within the interval  [ 0 , 0 . 5 ] . This figure shows $J(x^{T})$ versus *T*, *T* ∈ *T.*

*For *T* ∈ *T*, let $x^{T}$ denote the KSP policy generated by running the KSP algorithm on $\left(M_{T},10^{-6}\right)$. Fig 6 exhibits $J(x^{T})$, *T* ∈ *T*. It is observed that $J(x^{T})$ is increasing with the entended *T*.*

Based on 100 similar experiments, we conclude that the performance of the KSP algorithm is increasing with the extended knowledge-sharing promotion period. In practice, it is suggested to extend the knowledge-sharing promotion period to gain higher cost benefit of knowledge sharing.

Third, consider the influence of the maximum reward increase rate.

**Experiment 6.** *Let ${\bar{x}}^{*}$ be a vector generated randomly in the region $\prod_{i=1}^{N}(0,{\bar{x}}_{i})$. Let ${\bar{x}}_{1},\cdots \;,{\bar{x}}_{10}$ denote ten vectors obtained by scaling ${\bar{x}}^{*}$ such that ${\bar{x}}_{1}<{\bar{x}}_{2}<\cdots <{\bar{x}}_{10}$. Let $\bar{X}=\{{\bar{x}}_{1},\cdots \;,{\bar{x}}_{10}\}$. Consider the KSP instances*


$$M_{\bar{x}}=(N,T,\bar{x},f,g,\alpha ,c,P_{0}),\;\bar{x}\in \bar{X}.$$

**Fig 7:**
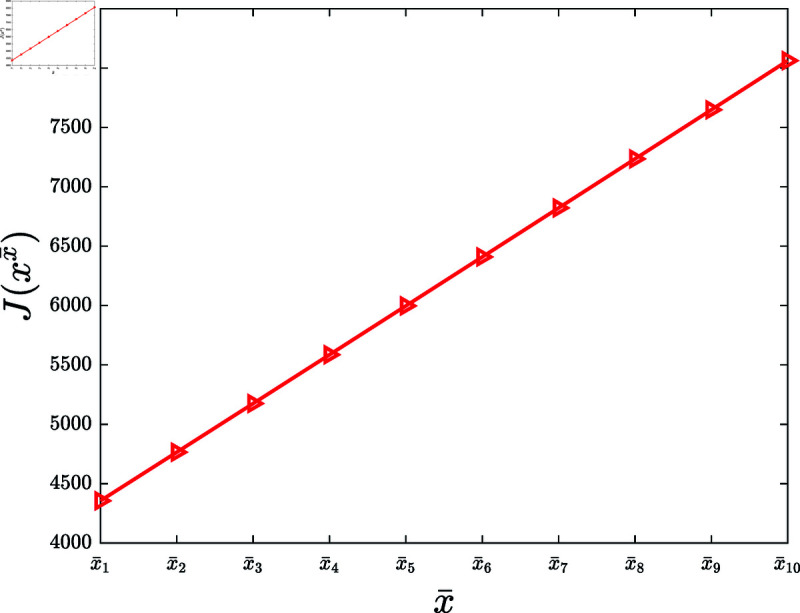
Experiment 6 is performed with the KSP instance $M_{\bar{x}}=(N,T,\bar{x},f,g,\alpha ,c,P_{0})$, where *N* = 100, *T* = 5, $\bar{x}\in \bar{X}$, *f* ( *z* ) = 0 . 3*tanh* ⁡  ( *z* ) , *g* ( *z* ) = 0 . 2*ln* ⁡  ( 1 + *z* ) , *α* = 0 . 05, *c* = 5, and $P_{0}$ is a vector generated randomly within the interval  [ 0 , 0 . 5 ] . This figure demonstrates $J(x^{\bar{x}})$ versus $\bar{x}$, $\bar{x}\in \bar{X}$.

*For $\bar{x}\in \bar{X}$, let $x^{\bar{x}}$ denote the KSP policy generated by running the KSP algorithm on $\left(M_{\bar{x}},10^{-6}\right)$. Fig 7 plots $J(x^{\bar{x}})$, $\bar{x}\in \bar{X}$. It is observed that $J(x^{\bar{x}})$ is increasing with the increasing $\bar{x}$.*

Based on 100 similar experiments. In each of these experiments, we conclude that the performance of the KSP algorithm is increasing with the increasing maximum reward increase rate. In practice, it is suggested to properly increase the reward budget to gain higher cost benefit of knowledge sharing.

Fourth, investigate the influence of the reward influence function.

**Experiment 7.** *Let $f_{1}(z)=0.21\tanh(z)$, $f_{2}(z)=0.22\tanh(z)$,  ⋯ , $f_{10}(z)=0.30\tanh(z)$. Let $F=\{f_{1},\cdots \;,f_{10}\}$. Consider the KSP instances*


$$M_{f}=(N,T,\bar{x},f,g,\alpha ,c,P_{0}),\;f\in F.$$

**Fig 8:**
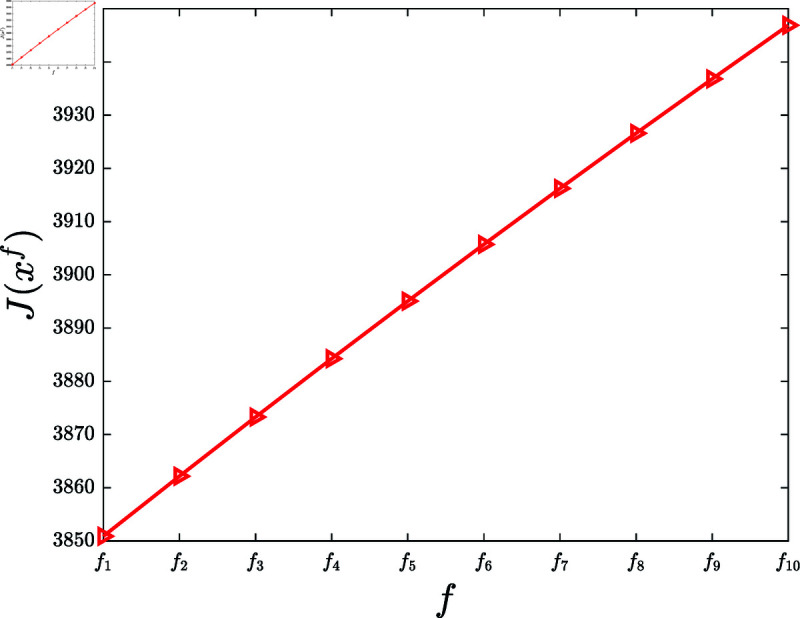
Experiment 7 is executed with the KSP instance $M_{f}=(N,T,\bar{x},f,g,\alpha ,c,P_{0})$, where *N* = 100, *T* = 5, $\bar{x}$ is a vector generated randomly in the region $\prod_{i=1}^{N}(0,{\bar{x}}_{i})$, *g* ( *z* ) = 0 . 2*ln* ⁡  ( 1 + *z* ) , *α* = 0 . 05, *c* = 5, and $P_{0}$ is a vector generated randomly within the interval  [ 0 , 0 . 5 ] . This figure depicts $J(x^{f})$ versus *f*, *f* ∈ *F.*

*For *f* ∈ *F*, let $x^{f}$ denote the KSP policy generated by running the KSP algorithm on $\left(M_{f},10^{-6}\right)$. Fig 8 depicts $J(x^{f})$, *f* ∈ *F*. It is observed that $J(x^{f})$ is increasing with the increasing *f*.*

Based on 100 similar experiments, we conclude that the performance of the KSP algorithm is increasing with the increasing reward influence function.

Fifth, look into the influence of the poster influence function.

**Experiment 8.** *Let $g_{1}(z)=0.11\ln(1+z)$, $g_{2}(z)=0.12\ln(1+z)$,  ⋯ , $g_{10}(z)=0.20\ln(1+z)$. Let $G=\{g_{1},\cdots \;,g_{10}\}$. Consider the KSP instances*


$$M_{g}=(N,T,\bar{x},f,g,\alpha ,c,P_{0}),\;g\in G.$$

**Fig 9:**
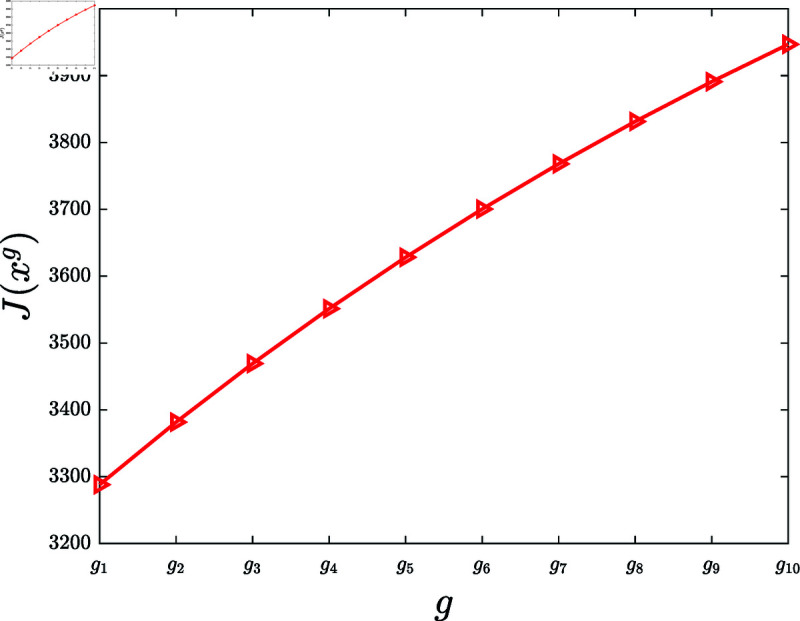
Experiment 8 is conducted with the KSP instance $M_{g}=(N,T,\bar{x},f,g,\alpha ,c,P_{0})$, where *N* = 100, *T* = 5, $\bar{x}$ is a vector generated randomly in the region $\prod_{i=1}^{N}(0,{\bar{x}}_{i})$, *f* ( *z* ) = 0 . 5*tanh* ⁡  ( *z* ) , *g* ∈ *G*, *α* = 0 . 05, *c* = 5, and $P_{0}$ is a vector generated randomly within the interval  [ 0 , 0 . 5 ] . The figure demonstrates $J(x^{g})$ versus *g*, *g* ∈ *G.*

*For *g* ∈ *G*, let $x^{g}$ denote the KSP policy generated by running the KSP algorithm on $\left(M_{g},10^{-6}\right)$. Fig 9 portrays $J(x^{g})$, *g* ∈ *G*. It is observed that $J(x^{g})$ is increasing with the increasing *g*.*

Based on 100 similar experiments, we conclude that the performance of the KSP algorithm is increasing with the increasing poster influence function.

Next, examine the influence of the lurking rate.

**Experiment 9.** *Let *A* = { 0 . 05 , 0 . 06 , ⋯ , 0 . 14 } . Consider the KSP instances*


$$M_{\alpha }=(N,T,\bar{x},f,g,\alpha ,c,P_{0}),\;\alpha \in A.$$

**Fig 10:**
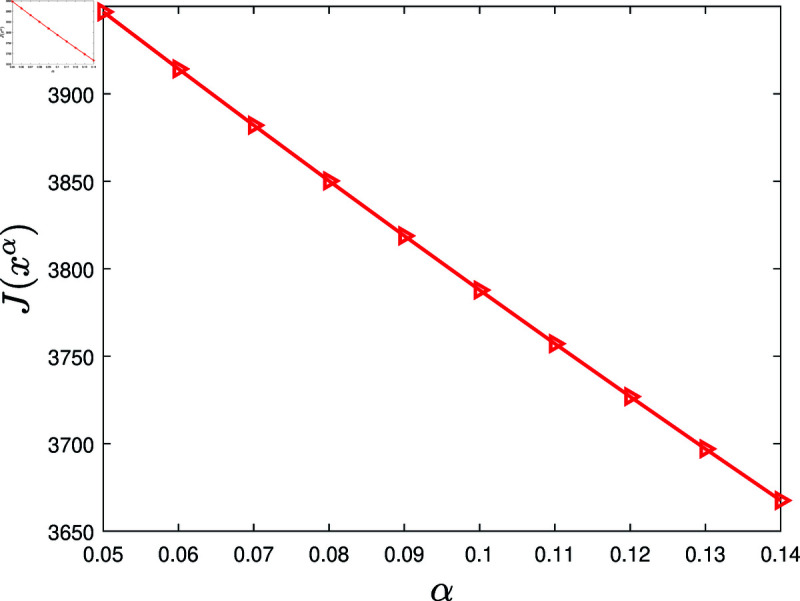
Experiment 9 is conducted with the KSP instance $M_{\alpha }=(N,T,\bar{x},f,g,\alpha ,c,P_{0})$, where *N* = 100, *T* = 5, $\bar{x}$ is a vector generated randomly in the region $\prod_{i=1}^{N}(0,{\bar{x}}_{i})$, *f* ( *z* ) = 0 . 3*tanh* ⁡  ( *z* ) , *g* ( *z* ) = 0 . 2*ln* ⁡  ( 1 + *z* ) , *α* ∈ *A*, *c* = 5, and $P_{0}$ is a vector generated randomly within the interval  [ 0 , 0 . 5 ] . The figure shows $J(x^{\alpha })$ versus *α*, *α* ∈ *A.*

*For *α* ∈ *A*, let $x^{\alpha }$ denote the KSP policy generated by running the KSP algorithm on $\left(M_{\alpha },10^{-6}\right)$. Fig 10 displays $J(x^{\alpha })$, *α* ∈ *A*. It is observed that $J(x^{\alpha })$ is decreasing with the increasing *α*.*

Based on 100 similar experiments, we conclude that the performance of the KSP algorithm is decreasing with the increasing lurking rate. In practice, it is suggested to take effective measures to stimulate lurkers.

Finally, examine the influence of the reward-return conversion coefficient.

**Experiment 10.** *Let *C* = { 5 , 6 , ⋯ , 14 } . Consider the KSP instances*


$$M_{c}=(N,T,\bar{x},f,g,\alpha ,c,P_{0}),\;c\in C.$$

**Fig 11:**
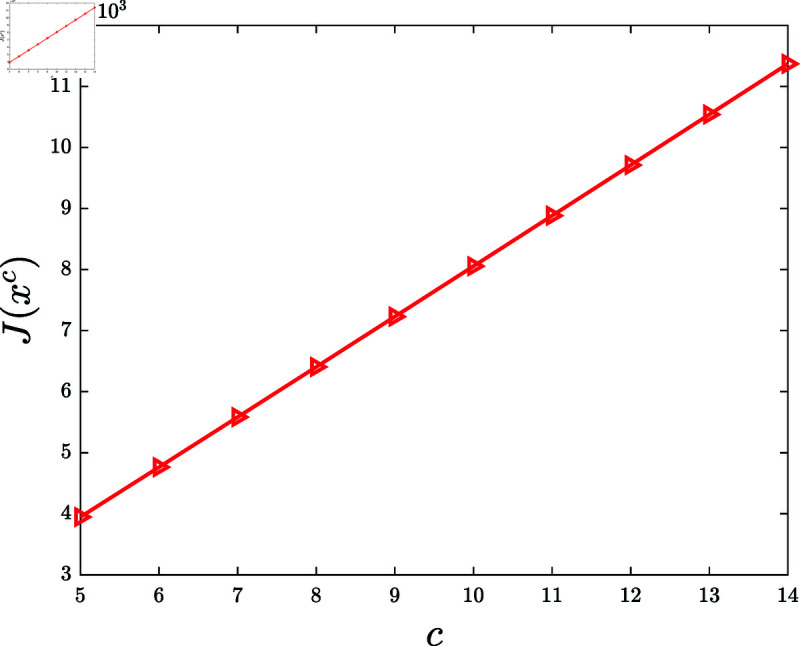
Experiment 10 is carried out with the KSP instance $M_{c}=(N,T,\bar{x},f,g,\alpha ,c,P_{0})$, where *N* = 100, *T* = 5, $\bar{x}$ is a vector generated randomly in the region $\prod_{i=1}^{N}(0,{\bar{x}}_{i})$, *f* ( *z* ) = 0 . 3*tanh* ⁡  ( *z* ) , *g* ( *z* ) = 0 . 2*ln* ⁡  ( 1 + *z* ) , *α* = 0 . 05, *c* ∈ *C*, and $P_{0}$ is a vector generated randomly within the interval  [ 0 , 0 . 5 ] . This figure depicts $J(x^{c})$ versus *c*, *c* ∈ *C.*

*For *c* ∈ *C*, let $x^{c}$ denote the KSP policy generated by running the KSP algorithm on $\left(M_{c},10^{-6}\right)$. Fig 11 demonstrates $J(x^{c})$ versus *c*, *c* ∈ *C*. It is observed that $J(x^{c})$ is increasing with the increasing *c*.*

Based on 100 similar experiments, we conclude that the performance of the KSP algorithm is increasing with the increasing reward-return coefficient.

In practice, the parameters *α* and *c* involved in the KSP model are roughly estimated, and the functions *f* and *g* involved in the KSP model are roughly approximated. As thus, the advantage of the KSP algorithm is questionable. Fortunately, it is observed from the above experiment results that the performance of the KSP algorithm depends continuously on the two parameters and the two functions. This observation implies that the KSP algorithm is robust to small perturbations of these factors. Consequently, the applicability of the KSP algorithm is further consolidated.

## Concluding remarks

This paper has proposed a novel knowledge sharing promotion problem. The problem has been reduced to an optimal control model. The model has been resolved successfully. We believe this work helps promote knowledge sharing.

There exist some open related issues. First, in this paper the knowledge-sharing community is assumed to be fixed. Due to the fluidity of organizational employees as well as the voluntariness of knowledge sharing, the knowledge-sharing community varies over time. Hence, it is worth studying the KSP problem for time-varying knowledge-sharing community [45,46]. Second, in this paper it is assumed that the employees’ response to monetary rewards is immediate. In practice, the response always lags behind the reward. In this context, the KSP problem should be modeled as a delayed optimal control problem [47,48]. Third, recent advances in adaptive reward mechanisms [49] suggest that dynamically adjusting reward intensity based on real-time participation levels—for instance, increasing incentives when lurkers dominate and reducing rewards when posters prevail—could further optimize cost-efficiency and long-term stability. Integrating such feedback-driven mechanisms into the KSP framework represents a promising direction. Next, in practice the monetary rewards for promoting knowledge sharing can only be awarded in a sequential manner. In this context, the KSP problem should be treated through optimal impulsive control approach [50,51]. Last, owing to the knowledge-sharing dilemma, there is interest conflict between the organization and a skilled employee. As thus, the KSP problem may be addressed through game-theoretic modeling [52–54].

## Supporting information

S1 Data Full data of Experiment 1. (XLSX)

S2 Data Full data of Experiment 2. (XLSX)

S3 Data Full data of Experiment 3. (XLSX)
